# Discovering the Learning Gradient of Students’ Preferences for Learning Environment

**DOI:** 10.3390/jintelligence11110206

**Published:** 2023-10-28

**Authors:** Carsten Kronborg Bak, Simon Schulin, Jeanne Krammer

**Affiliations:** Department of Social Work, University College of Southern Denmark, 6700 Degnevej, Denmark; ssch@ucsyd.dk (S.S.); jokr@ucsyd.dk (J.K.)

**Keywords:** online teaching, learning approaches, self-regulation, meta cognitive, cluster analysis, learning gradient, COVID-19 lockdown

## Abstract

The aim of this study was to examine the effects of online learning self-regulation on learning outcomes during the COVID-19 pandemic lockdown among university college students. Quantitative k-means cluster analysis was used to examine the relationship among students in three different clusters based on their preferences toward online learning. The results indicated that online learning self-regulation had a significant positive effect on learning outcomes due to the shift to online learning. Thus, we identified a “learning gradient” among students, showing that cluster 1 students (preferences for 100% online) have the most positive preferences toward online teaching and the highest degree of self-regulation and learning outcome, cluster 2 students (moderate preferences for both physical and online teaching) are mixed (both positive and negative experiences) and moderate self-regulation and learning outcomes while cluster 3 students (preferences for physical classroom teaching) have the most negative preferences and the lowest self-regulation and learning outcome. The results from this study show that students’ self-regulated learning strategies during online teaching environments are important for their learning outcomes and that cluster 1 and 2 students especially profited from the more flexible online learning environment with organized and deep learning approaches. Cluster 3 students need more support from their educators to foster their self-regulation skills to enhance their learning outcomes in online teaching environments.

## 1. Introduction

The COVID-19 pandemic has altered our lifestyles, work patterns, and educational approaches. With the sudden worldwide closure of higher educational institutions in the spring of 2020, we all had to adapt to online learning environments and platforms (Marinoni 2020; Parpala et al. 2021). Although online learning has been an alternative mode of education for some time, it has never been implemented on a large scale before the pandemic. Many students have reported feeling overwhelmed, isolated, and disengaged from their learning (Uslu Kocabaş and Bavlı 2021). Students and teachers had to learn new technological tools, adjust to different modes of instruction, and find ways to stay motivated and engaged in their learning. A huge amount of the COVID-19 research has emphasized the negative effects of the shift in the learning environment on students’ degree of learning engagement (Huckins et al. 2020; Petillion and McNeil 2020) and students’ reduced mental well-being and social contacts (Huckins et al. 2020; Kaparounaki et al. 2020; Wang and Zhao 2020; Van Eekert et al. 2023).

At the same time, the pandemic has provided an unexpected opportunity to examine the impact of self-regulation on online learning outcomes among students, partly due to the possibility of isolating subjective learning strategies from social-relational classroom teaching. Online learning has required students to take more responsibility for their learning, and this may have been particularly challenging for students who have not yet developed strong self-regulatory skills. Studies conducted by Zimmerman (2002), Zimmerman and Kitsantas (2014), Zimmerman and Labuhn (2012), Biggs (1987), and Zimmermann et al. (2020) have demonstrated the significance of students’ ability to self-regulate their learning. These studies highlight that self-regulation is an active, proactive engagement by students in their own learning process. It can be creating a strategy yourself rather than passively waiting for a teacher’s instructions.

Intelligence, self-regulation, and learning are inherently interconnected in the term intelligence, despite “intelligence-school”, collective intelligence, g-factor, personality traits/motivational factors, and multiple intelligence (Oliwa 2022; Morosanova et al. 2022; Rowe et al. 2021; Deary et al. 2007).

We refer to CHC structural models of intelligence that offer a comprehensive framework for understanding the multifaceted nature of cognitive abilities. These models, developed by Cattell, Horn, and Carroll, categorize intelligence into broad domains (e.g., fluid, crystallized, and memory) and specific abilities (e.g., verbal comprehension and working memory), allowing a nuanced assessment of cognitive strengths and weaknesses (Schneider and McGrew 2012). For students, this framework holds substantial relevance, as it provides educators with insights into diverse learning approaches/styles and intellectual profiles. By tailoring teaching methods to individual cognitive strengths, educators can enhance student engagement and comprehension. Moreover, CHC models can inform strategies for self-regulated learning. Students who understand their cognitive profile can optimize their study approaches, focusing on their strengths while developing weaker areas. In essence, CHC models empower students to take ownership of their learning journey through informed decision making and effective self-regulation strategies (McGrew and Wendling 2010).

Metacognition refers to the ability to think about and regulate one’s own thinking processes, and it involves being able to understand one’s own thoughts, knowledge, and learning strategies (Zimmerman 2000; Flavell 1979; Pintrich 1999). As Anthonysamy (2021) points out, metacognition is crucial to students’ talent for learning on their own but is lacking among university students. Many students were surprisingly not equipped with the relevant skills to perform in online learning despite being used to technology. They are not aware of how to examine how they learn and how to judge which methods are effective when faced with new forms of learning online (Anthonysamy et al. 2020). 

Based on a mixed-method research design, the authors initially conducted a quantitative cluster analysis followed by five qualitative focus group analyses to explore university college students’ experiences with online teaching one year after the COVID-19 lockdown in Spring 2020. The qualitative findings have been published in an article titled “University College Students’ Experiences with Online Teaching One Year after COVID-19 lockdown in Spring 2020” (Bak and Schulin 2023). Utilizing k-means cluster analysis, we first identified three distinct student clusters based on their preferences for online teaching (see method section). Subsequently, we employed the quantitative analysis results to discern overarching themes, including learning approaches, perceived learning outcomes, study planning, and both positive and negative experiences, included in five in-depth focus group interviews involving 29 students from diverse departments and educational backgrounds at University College South Denmark (Bak and Schulin 2023).

The qualitative findings underscore the significance of students’ self-regulated learning strategies within the online teaching environment and bring to light a notable disjunction between learning outcomes and the conventional classroom as a fixed learning environment. This research offers valuable insights into the nuanced and multifaceted experiences of students in the realm of online education, elucidating the various complex factors that influence their learning outcomes (Bak and Schulin 2023).

This article presents a detailed examination of our quantitative cluster analysis and shift in students’ learning environments in the period from 2018 to 2021 to examine if the shifts influence students’ learning approaches and learning outcomes. Furthermore, we delve deeper into our qualitative research findings, focusing on the interplay between students’ learning approaches, self-regulated learning skills, and metacognitive learning processes. Additionally, the CHC structural model of intelligence provides a comprehensive framework for understanding the diverse cognitive abilities associated with enhancing students’ learning. By exploring these dynamic relationships, we can gain valuable insights into how intelligence manifests and influences learning outcomes in online environments (Artelt et al. 2003; Schraw and Moshman 1995), thereby informing educational practices and optimizing online learning experiences for students.

In the realm of research on self-regulated learning, the focus extends beyond individual students’ learning preferences and strategies. It encompasses social aspects of learning, such as seeking assistance from peers and receiving feedback from instructors. Self-regulation predominantly revolves around self-motivation and metacognitive processes, as emphasized by Zimmerman and Kitsantas (2014).

However, in online learning environments, students may have less structure and support, and they may need to rely more on their own self-regulatory skills to succeed. Their own learning is more likely to set achievable goals, monitor their progress, and adapt their strategies to meet their goals. Self-regulated learners are also more motivated, have higher self-esteem, and are better able to cope with academic challenges (Zimmerman and Schunk 2011). 

Numerous studies indicate that online teaching, whether in the form of blended learning or distance learning, offers significant learning benefits. This holds true for both perceived and actual learning outcomes, as highlighted by Joksimovic et al. (2015), Ellis et al. (2017), and Bernard et al. (2014). While the magnitude of the learning effect may not be substantial in some cases, it remains statistically significant (Bernard et al. 2014). According to these studies, one contributing factor to this effect is the ability of students to access teaching materials flexibly. This allows students to learn at their own pace and revisit relevant sections of the content when necessary. Furthermore, online teaching’s flexibility and content cater to diverse learning preferences and approaches, thereby enhancing students’ learning opportunities (Bak and Schulin 2023; Truong 2016; Khaddage et al. 2015).

Simultaneously, research demonstrates that the effectiveness of online teaching depends on how it is organized and implemented. Merely incorporating digital technology into education does not automatically yield positive outcomes. Transforming conventional teaching into an online format to achieve the intended outcomes is a more complex process than it may seem (Means et al. 2013; Khaddage et al. 2015). In fact, numerous studies highlight that very little can be directly transposed from traditional classroom instruction to online teaching.

To have a beneficial impact on students, everything must be developed and executed using distinct methods and formats. In essence, it is the approach or methodology employed in online teaching that influences its effectiveness rather than the mere utilization of technology in instruction (Rambøll 2016).

Our perspective aligns with the findings of several researchers, including Parpala et al. (2021), Utama et al. (2020), Parpala et al. (2013), and Richardson and Price (2003), who have noted that prior studies have predominantly focused on assessing students’ experiences and well-being while neglecting the importance of understanding students’ learning approaches and study preferences. Research suggests that students’ study methods and learning strategies are closely intertwined with their attitudes—whether positive or negative—toward the new online teaching–learning environment and their capacity to self-regulate their studies. Dabbagh (2007) has further suggested that successful online learners should possess interpersonal and communication skills, in addition to a strong academic self-concept, to effectively apply self-regulated learning strategies.

There is a well-established research tradition focused on student approaches to learning (SAL) in Europe and other parts of the world, particularly within higher education. This empirical tradition has been explored in various studies conducted by scholars such as Biggs (1987), Entwistle and Ramsden (1982), Trigwell et al. (1999), Evans (2014), and Lindblom-Ylänne et al. (2019).

This article aimed to explore the effects of online learning self-regulation on learning outcomes during the COVID-19 pandemic using k-means cluster analysis. The analysis will help to identify patterns of self-regulation among university college students divided into three clusters and examine the relationship between students’ learning approaches, self-regulation, and learning outcomes. The following research question was examined in a longitudinal design from 2018 to 2021: *What is the relationship between university college students’ learning approaches, self-regulation, and learning outcomes in physical classroom teaching and the shift to online learning environments during the lockdown in 2020*?

## 2. Materials and Methods

In this study, we used survey data collected from university college students from different educations and institutes at University College South in Denmark. Data from a survey following the COVID-19 pandemic were used to examine the relationship among students in three different clusters based on their preferences toward online learning. We employed the “HowULearn” questionnaire (LEARN), designed to assess various learning approaches among students (Parpala and Lindblom-Ylänne 2012). This questionnaire was utilized to investigate the experiences of university college students with online teaching both before, during, and after the lockdown in spring 2020.

Furthermore, a possible correlation was investigated between preference toward online teaching and how students assessed the importance of social relations and activities in relation to their academic learning outcomes from teaching.

### 2.1. Data from National Dataset during COVID-19

UC SYD participated as one of nine higher education institutions in a survey regarding the experience of online teaching during the COVID-19 pandemic lockdown (Georgsen and Qvortrup 2021). The criteria for inclusion were enrollment in a course and received teaching during the COVID-19 lockdown. Students who were on leave during the lockdown and exchange students were excluded from the survey. A total of 1316 students completed the survey, which corresponds to a response rate of 20.6% (Table 1).

### 2.2. Data from LEARN Surveys

The data from LEARN were collected in three waves. The first wave was before the lockdown in 2018 (N = 487). The next was during the early stages of the lockdown in spring 2020 (N = 3747) and again in spring 2021 (N = 2548) after returning not exactly to “normal”—but rather blended learning provision in the physical classroom teaching at the campus.

To examine the experiences of university college students with online teaching before, during, and after the spring 2020 lockdown, we employed the LEARN questionnaire, which was developed by Finnish researchers Parpala and Lindblom-Ylänne in 2012. The LEARN questionnaire measures students’ different approaches to learning (deep approach, organized approach, and unreflective/surface approach) and indicators of the teaching–learning environment (e.g., peer support, constructive feedback, and motivational teaching). It is possible to see the questions in the LEARN questionnaire using this link: https://blogs.helsinki.fi/howulearn/files/2012/12/HowULearn-2016-1_English-1.pdf (accessed on 28 June 2023).

The LEARN questionnaire focuses on students’ learning and their learning approaches, whether it concerns physical classroom teaching or online teaching during the lockdown. Therefore, it is an adequate and valid questionnaire where we can compare the results of student’s experiences with the same scale and questions during shifts in different learning environments in the period 2018–2021. The Likert scale was used to measure the questions with a range score from 0 to 5. 

SPSS (Statistical Package 27) was used for the statistical analysis.

Ethical statement

The data utilized in this article were obtained from previous surveys, where all participants provided informed consent for their inclusion prior to participating. The study adhered to the principles of the Declaration of Helsinki and received approval from the Ethical Committee of University College South of Denmark (Project code: 2023-3).

### 2.3. Statistical Analyses

Initially, post-stratification weights were made for the dataset following the COVID-19 lockdown due to an expectation that the respondents and the population would differ from each other due to non-response. Poststratification weights were calculated using the propensity scores approach (Li et al. 2018), and the weights were calculated based on the population’s distribution by gender, age, origin, qualifying education, and institute. 

Descriptive statistics were used to present the sociodemographic characteristics of the respondents, and furthermore, cluster analysis was used to organize respondents into clusters based on how closely associated they are in preference for online teaching. There are several methods often used to cluster data, including k-means clustering. K-means is a centroid-based algorithm that aims to partition the respondents into k clusters so that the similarity between respondents in one cluster is high while the level of similarity to the respondents in other clusters is low (MacQueen 1967). In preparation for the cluster analysis, relevant comparison variables are selected, which, in this instance, are variables concerning preferences for online teaching. Table 2 shows the questions used in the K-means clustering.

There are generally two types of cluster formation. We used the non-hierarchical k-means method (Hussain and Lauridsen 2017), which is characterized by the fact that the number of clusters to be formed is determined in advance. Here, SPSS selects random respondents as starting clusters and then assigns the other respondents to the cluster they are closest to. However, one must be aware that when the result of the cluster analysis depends on which respondents are selected for “starting clusters”, this can affect the reliability (Bak and Schulin 2022). Three clusters were determined, the number being tested via the hierarchical cluster analysis (ward’s minimum variance method was used). Here, clusters of 6, 5, 4, and 3 were analyzed, and a clear picture emerged of a cluster that strongly agreed on comparison variables and a cluster that strongly disagreed on comparison variables. Therefore, for interpretative reasons, three clusters were chosen with the assumption that there will be a cluster with a preference for online teaching, a cluster that prefers physical attendance teaching, and a cluster that oscillates between slightly agree/disagree according to comparison variables.

An exploratory factor analysis and a reliability analysis were performed on the four different indicators for self-regulated learning, indicating the four items can represent one factor (Cronbach’s α = 0.7). Similar statistical analyses were performed on the three different indicators for learning outcomes (Cronbach’s α = 0.8), and the three items can be grouped into one factor. The scales for self-regulated learning and learning outcomes were divided into two groups using a median split, enabling bivariate analyses. Participants scoring at or below the median were categorized as having “limited” self-regulated learning or learning outcomes, while those scoring above the median were categorized as having “sufficient” self-regulated learning or learning outcomes.

Table 3 shows the questions used for the analysis of the factors: Learning outcome and self-regulated learning.

Additionally, we conducted bivariate analyses employing the chi-squared test to investigate potential differences, including preferences for online teaching, among the two levels of self-regulated learning and learning outcomes.

A *t*-test was carried out with the aim of investigating possible differences in the mean value for the learning environments and learning approaches measured in the years 2018, 2020, and 2021 (LEARN). Data from LEARN 2021 also contain supplementary exploratory questions regarding preference for online teaching, which are compared with background characteristics, learning approaches, and the importance of the social/relational (regardless of whether it is physical or online) for one’s professional learning outcomes (Bak and Schulin 2022).

For all analyses, a significance level of *p* < 0.05 was considered. Given the potential for large sample sizes to yield statistically significant associations with negligible practical importance, the strength of the associations was evaluated using Cramér’s V for the chi-squared test and Cohen’s d for the *t*-test. For Cramér’s V, an effect size is considered small at ≥0.1, medium at ≥0.3, and large at ≥0.5. For Cohen’s d, an effect size is deemed small at ≥0.2, medium at ≥0.5, and large at ≥0.8 (Cohen 1988). When interpreting the results, a value of Cramér’s V below “small” implies that the difference is practically negligible despite being statistically significant.

## 3. Results

**Figure 1:**
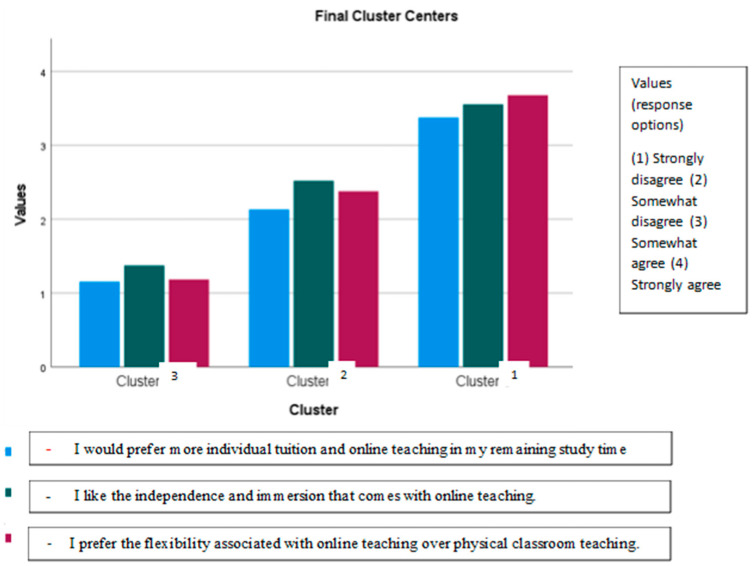
Cluster profiles. N = 880 *. Mean values. * Some students are excluded due to the answers “don’t know” and “not relevant” (*n* = 436).

In this section, we present the main results from our cluster analysis to examine our research question of the relationship between university college students’ learning approaches, their self-regulation, and learning outcomes in the shift to online learning during the COVID-19 lockdown. The cluster profiles based on the three comparison variables measuring preference for online teaching are presented in Figure 1. 

The cluster profiles, based on the comparison variables (Table 2), are characterized by the preference for online teaching. Cluster 3 is characterized by preferring physical classroom teaching (43.1%), cluster 2 oscillates between slightly agree/slightly disagree according to comparison variables (25.8%), and cluster 1 is characterized by preferring online teaching (31.1%); see Figure 1. ANOVA analyses show that the clusters are significantly different in the mean values for the comparison variables (*p* < 0.001). As further validation of the cluster analysis, discriminant analysis is carried out with the three clusters as the dependent variable and comparison variables as independent variables. Based on comparison variables, 99% of the respondents can be classified into the correct clusters.

Using data from a national dataset (N = 880), we examined our research question about the relationship between students’ learning approaches, self-regulation, and learning outcomes. We identified what we call a “learning gradient” showing that cluster 1 students have higher percentage of students with a high degree of learning outcome (81. 4%) and self-regulation (72.6%), cluster 2 students have moderate results with a high learning outcome of (61.2%) and self-regulation on 45.4%, and cluster 3 students have the lowest degree of learning outcome (33%) and self-regulation (26.9%) (Bak and Schulin 2023). See Table 4.

**Figure 2:**
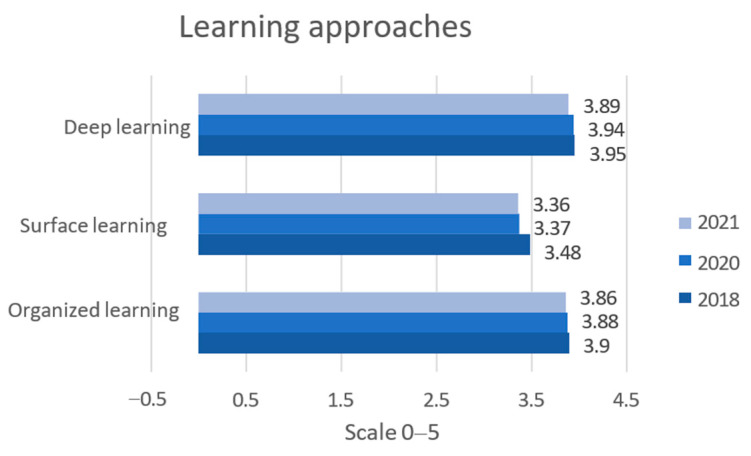
Learning approaches among university college students before (2018), during (2020), and one year after COVID-19 lockdown in 2021. Source: LEARN 2018 (N = 487), 2020 (N = 3747), and 2021 (N = 2548).

We examined student’s different approaches to learning during “normal” physical teaching in the classroom in 2018, during lockdown in 2020, and again returning to “normal” classroom teaching in spring 2021 (Figure 2). The overall result shows no significant difference in the learning approach “organized learning” over the years. However, the observed difference was determined to be of minimal practical significance (*p* = 0.002, d < 0.2).

**Figure 3:**
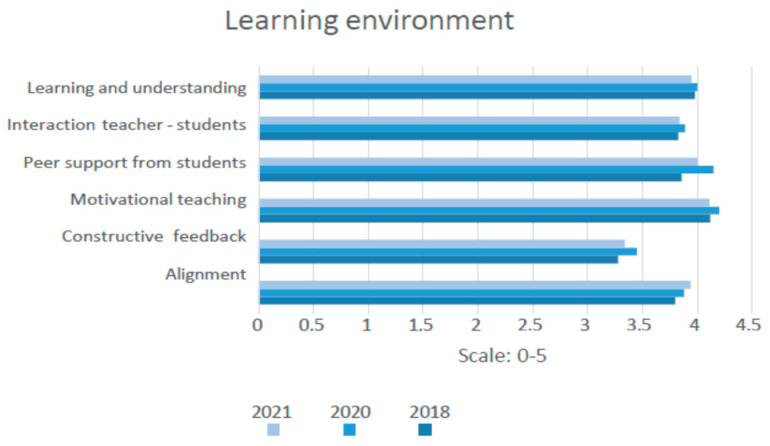
Shift in learning environment before, during, and after COVID-19 lockdown, 2018–2021. Source: LEARN 2018 (N = 487), 2020 (N = 3747), and 2021 (N = 2548).

Figure 3 shows minor increases and decreases in the shift from physical classroom teaching (2018) to online teaching (2020) and back to “normal” classroom teaching in 2021. Even though statistically significant differences are found in the indicators for the learning environment, the differences are found to be practically negligible (*p* < 0.001, d > 0.2).

Table 5 shows that university college students seemingly do not have the same need for social relations and activities in relation to their academic learning outcomes from teaching, regardless of whether it concerns physical or online teaching. The variable “Impact of social activities on students learning outcome”, which is constructed as a scale from 1 to 10, was divided into low importance (1–4), medium importance (5–7), and high importance (8–10). Students with a preference for physical classroom teaching assess the social/relational element of greater importance to their academic outcomes than students with a preference for a combination of physical attendance and online teaching and students preferring only online teaching. 

## 4. Discussion

The aim of this article was to examine the relationship between university college students’ learning approaches, their self-regulation, and learning outcomes in physical classroom teaching and the shift to online learning environments during the COVID-19 lockdown. The data interpretation was possible due to the emergence of the new reality with a global shift to online teaching, thus pathing the way to isolate the individual learning approach from the physical classroom into a matter of an analysis of the subject’s self-regulation. 

### 4.1. Comparison of Our Quantitative and Qualitative Results

In this section, we compare our quantitative findings with our previously published qualitative results (Bak and Schulin 2023) and discuss our results with existing research in this research domain. Together, these mixed method results make unique insights. First, we identified or “discovered” what we call a “learning gradient” among university college students in an online learning environment. This gradient reveals that students in cluster 1 exhibit the most positive preferences toward online teaching, along with the highest levels of self-regulation and learning outcomes. Cluster 2 students have mixed experiences, with both positive and negative aspects, moderate self-regulation, and learning outcomes. On the other hand, cluster 3 students demonstrate the most negative preferences, lowest self-regulation, and lowest learning outcomes (Table 4). Consequently, we believe it is crucial to discuss the significance of students’ metacognitive learning strategies, their preferences toward physical or online teaching environments, and the influence of their learning approaches in more detail in the following sections.

The quantitative cluster results showed in detail a closer connection between students’ degree of self-regulation and their learning outcomes in the three clusters and that the shift in learning environments in the period 2018 to 2021 does not affect, or only slightly affects, students’ learning approaches. In both studies, we find self-regulated learning strategies crucial. The contribution of the qualitative article (Bak and Schulin 2023) was to show important variation in and between the three clusters, trying to find variation, inconsistencies, and more complex explanations for differences in students’ self-regulation and learning outcomes. For instance, the qualitative results from focus group interviews showed that some students were good at planning and self-regulating their study efforts and appreciated the high flexibility in online teaching, although they at the same time could mention all the challenges related to the quick shift to online teaching during the COVID-19 lockdown. On the other hand, other students (mostly cluster 3) experienced that it was very difficult to plan and self-regulate their study and often referred to the responsibility of the teacher and what they were used to doing in the physical classroom teaching before the lockdown. 

The qualitative results highlighted the overlap between the three clusters, showing that it is difficult to obtain strict borders—e.g., cluster 1 students with high self-regulation and learning outcomes also emphasize the importance of social contacts to their study mates even though it is not regarded to be as important for them as cluster 3 students. Cluster 2 students perhaps represent the overlaps best as they mostly refer to both “pro and cons” when discussing their experiences with online teaching. The discussion between students in the three clusters also showed that, e.g., even some of the most critical cluster 3 students would agree on the positive aspects of more flexibility during online teaching during the lockdown, and most students could agree on positive changes from the lockdown (e.g., more time to read on your own, and supervision online and group work on teams) are something they still appreciate after returning to “normal” teaching again.

However, we must take our selection criteria into consideration for participation in focus group interviews. Most students (17 students) were basically against online teaching; seven preferred more online teaching, and five had mixed experiences of both advantages and disadvantages (Bak and Schulin 2023). This might have influenced the discussions and opinions of participants in the focus group interviews by dominating views by majority vs. minority constellations. Another aspect concerns the timing of the focus group interviews in spring 2021, which might influence both the experiences and views one year after the first lockdown.

### 4.2. Metacognition and Students’ Learning Strategies

Metacognitive models of self-regulation prominently feature metacognitive monitoring and control processes. This entails that self-regulated learners are accustomed to assessing and monitoring their ongoing learning progress (metacognitive monitoring) and making informed decisions regarding their priorities and approach (metacognitive control). They possess knowledge about effective strategies and apply these strategies in their learning endeavors (Rea et al. 2022).

We observed that this aligns well with the outcomes, particularly for students in cluster 1 and, to some extent, cluster 2. However, it is worth noting that a significant portion of the cognitive literature commonly characterizes learners as lacking both the metacognitive knowledge required to assess the effectiveness of learning strategies and the ability to monitor their own learning progress (Lawson et al. 2019) and, therefore, make suboptimal self-regulated learning decisions (Rea et al. 2022). We believe that this is what we found among cluster 3 students who have the most difficulties in changing to an online learning environment during the COVID-19 lockdown. Their self-regulation was low, and they had the most negative attitudes toward online teaching, leading to a more passive engagement and academic achievement with the lowest learning outcomes.

Cluster 3 students seem to lack these metacognitive strategies and often (in focus group interviews) refer to teachers having the responsibility to plan or regulate their learning efforts on their behalf (Bak and Schulin 2023). These students seem to lack self-awareness and understanding of their own thought processes through self-reflection utilized by planning, monitoring, and regulating strategies (Anthonysamy 2021; Anthonysamy et al. 2020). It seems relevant to ask whether many years of teacher-directed learning in the physical classroom bear responsibility for this. 

### 4.3. The Importance of Students’ Learning Approaches

Research on students’ preferences and learning outcomes in online teaching has gained considerable attention, particularly with the widespread adoption of online learning during the COVID-19 pandemic. However, despite the rapid evolution of online research, there is still a dearth of studies that comprehensively incorporate students’ learning approaches (Parpala et al. 2021).

The early contributions from various authors of learning approaches in the 1970s and 1980s about how people learn differently (deep or surface learning) are still highly relevant for today’s research on learning (Urhahne 2020; Biggs 1987; Entwistle and Ramsden 1982), which is also the case for the online learning environment. As Urhahne (2020) points out, differences occur when people work on the same learning task; they differentiate between a surface and a deep learning approach, and the key message from learning models was that the pursued approach to learning is a decisive factor in learning outcomes. 

Our findings, which analyzed students’ transition from traditional classroom instruction to online teaching between 2018 and 2021, indicated that, overall, students did not significantly alter their learning approaches. However, these results did emphasize the strong correlation between students’ learning approaches and their preferences for either a physical or online teaching environment, as highlighted by Bak and Schulin (2023). Cluster 1 and 2 students exhibited similarities to previous research on “organized” and “deep approach students” (e.g., Parpala et al. 2010; Asikainen and Katajavuori 2022; Parpala et al. 2021; Kuittinen and Meriläinen 2011; Kyndt et al. 2011; Trigwell et al. 2012). However, it is worth noting that our cluster 2 students displayed mixed experiences, both positive and negative, underscoring the importance of considering students’ preferences when evaluating learning outcomes in online teaching (Bak and Schulin 2023).

### 4.4. Learning Approaches and the Ability to Self-Regulate Their Study Efforts

According to Rogers and Swan (2004) and Zimmerman (2002), self-regulated learning is defined as learners’ ability to control and manage their own learning process, encompassing behavior, cognition, and motivation consciously and actively. Despite numerous research studies demonstrating the positive impact of self-regulatory processes on academic success, it is worth noting that a limited number of teachers presently equip their students with the skills needed for independent learning, as highlighted by Zimmerman (2002).

Cluster 3 students in our research apply the unreflexive (surface) approach to learning. They have negative perceptions of the online teaching–learning environment, experience heavier workloads, and lower self-regulation skills. This is also studied among students with a surface approach in more detail (e.g., Lindblom-Ylänne et al. 2019; Govaerts et al. 2011). This research also mentions the lack of capabilities to organize their own study in an online learning environment, missing the teacher as an instructor, and too many challenges, or too few, when studying has been shown to steer students toward applying the surface approach.

Parpala et al. (2021) emphasized the significance of recognizing that students categorized within the unorganized and surface profile, like our cluster 3 students, reported the most unfavorable experiences within the teaching–learning environment. They also achieved the lowest scores in deep approach and organized studying, consistent with previous research findings that an unreflective (surface) approach is inversely associated with positive experiences in the online teaching–learning setting (Herrmann et al. 2017; Parpala et al. 2013; Richardson and Price 2003). Asikainen and Katajavuori (2022) also highlighted that certain students, particularly those in cluster 3, encountered challenges related to “taking responsibility” for time management and struggled with procrastination.

### 4.5. Social Interaction Important—But Not Equally for All Students

Our results show that social interaction is regarded as important for university college students, whether it concerns physical or online teaching. Table 5 shows that 67% of the university college students (cluster 3) found social activities and relations highly important for their learning outcomes, but only 32% among cluster 1 students found them important for the highest learning outcomes. This result emphasizes the importance of how we discuss the impact of social activities/relations during the pandemic. It is important to mention that only 20% of cluster 1 students mentioned the “low” impact of social activities/relations on learning outcomes.

The discussion in most of the research literature, however, concerns a more general discussion of how the rapid shift to online teaching during the pandemic has disrupted the “normal” everyday life of students and affected their well-being, some students being more socially isolated and missing the normal teacher–student relation from the physical classroom (Georgsen and Qvortrup 2021). Pavin highlights the fact that students who miss out on typical academic interactions tend to encounter greater challenges in terms of learning and self-regulation within an online learning environment (Pavin Ivanec 2022). He also cites previous studies that link these challenges to the adverse effects of disruptions caused by the pandemic, such as increased stress, diminished well-being, and social isolation.

The results from Table 5, however, show the importance of dividing students into different clusters due to their preferences toward the online learning environment and their learning approaches to obtain a more differentiated picture of the impact of social activities/relations during the pandemic. The picture is quite clear as even cluster 1 students find social activities of middle or high importance even though their learning outcomes are high and could indicate very deep and independent learning. Perhaps this result could be interpreted as a well-established narrative from many years of physical teaching in the classroom (Bak and Schulin 2023). 

### 4.6. A Need for More Support Online for Some Students

We recognize the importance of prioritizing students’ well-being in the realm of online learning. In this regard, Parpala et al. (2021) advocate for the use of a tool rooted in acceptance and commitment (ACT) intervention. This tool is designed to enhance students’ psychological flexibility, promote academic progress, and alleviate challenges related to studying (Asikainen et al. 2018; Parpala et al. 2021).

This approach appears to be particularly relevant for cluster 3 students, as it can help them develop study skills online and assist in organizing their learning. Providing explicit instructions and guidance on structuring and planning their learning can foster their study skills (Bak and Schulin 2023; Holzer et al. 2021). The traditional teaching approach likely fosters expectations where the teacher assumes the most significant role, potentially leading to reduced self-regulation and responsibility for one’s own learning.

### 4.7. Future Research

We recommend that future research give priority to exploring how the development of asynchronous learning activities can enhance students’ self-regulated learning strategies within the online learning environment. This research should also seek to gain insights into the various learning approaches of students and their capacity to self-regulate their studies. Moreover, we concur with the findings of Katajavuori et al. (2021), which suggest that the implementation of an ACT-based course in higher education can support the well-being and study skills of students, particularly those in cluster 3, resulting in more effective learning.

Additionally, conducting further cluster analysis to examine how student learning outcomes and self-regulation vary based on factors such as educational level, study program, and age would be valuable. Such analysis can offer insights into the challenges faced by different student groups during the 2020 COVID-19 shutdown and help identify strategies to better assist these students (Bak and Schulin 2023).

### 4.8. Limitations

There are some limitations of our study. First, it is relevant to consider measurement validity. Although we find the validity of the LEARN questionnaire and the indicators of “learning approaches” and “learning environment” high, it is difficult to measure constructs, such as self-regulation or deep learning, that are complex and multidimensional to capture all relevant aspects with a single instrument. We tried to take account of this challenge with our qualitative focus group interviews (Bak and Schulin 2023) that contributed a more detailed picture of students’ learning approaches. Second, in our quantitative research, we must rely on predefined variables and measurement instruments, which may not capture the full range of learning approaches and self-regulation strategies. This could lead to oversimplification of these complex constructs. We believe that our qualitative results complement our quantitative cluster results with more nuanced and contextual aspects that could have an impact on the variables we use from the LEARN questionnaire.

Third, we conducted our research among students in University College South (UC SYD), and therefore, our aim was not to be able to generalize the results of the study but to examine the shift from physical to online learning among students from the same institution in the period of 2018 to 2021.

Additionally, we find it crucial to consider students who did not provide responses in the LEARN questionnaire during the period from 2018 to 2021, as highlighted by Bak and Schulin in 2023. Moreover, we recognize the potential for a more comprehensive exploration of variances among disciplines in terms of their learning approaches and preferences for online teaching environments, as indicated by Parpala et al. (2010).

### 4.9. Conclusions

In conclusion, rather than advocating a return to traditional classroom teaching, we emphasize the importance of considering each student’s unique learning approach when designing online courses. Often, online courses are developed with a one-size-fits-all approach, mimicking physical classroom instruction (Bak and Schulin 2023). Our focus group interviews revealed a diverse range of needs among university college students. It is possible to create online courses that better cater to these varying learning approaches, offering more personalized instruction than traditional classroom settings, as supported by Bak and Schulin (2023) and Navarro and Shoemaker (1999). The integration of the learning gradient into future educational organizations holds the potential to enable all students to thrive in their learning journeys.

## Figures and Tables

**Table 1:** Characteristics of the study population. Percent.

Characteristics	Population (*n* = 6386)	Respondents (*n* = 1316)	Respondent Weighting (*n* = 1316)
Age (years—mean)	28.16	29.46	28.17
Women (%)	76.5%	83.8%	76.7%
Danish origin (%)	88.9%	86.9%	88.6%
Qualifying education—general high school (%)	33.0%	34.8%	32.8%
Institute			
Education	19.4%	18.9%	18.2%
Pedagogue	30.5%	28.7%	28.8%
Society and administrations	20.6%	19.7%	20.4%
Health	29.5%	32.7%	30.6%

**Table 2:** Questions and response options.

Questions	Response Options
Q: How much do you agree or disagree with the following statement: - I would prefer more individual tuition and online teaching in my remaining study time. - I like the independence and immersion that comes with online teaching. - I prefer the flexibility associated with online teaching over physical classroom teaching.	The response options were on a four-point Likert scale: (1) strongly disagree, (2) somewhat disagree, (3) somewhat agree, and (4) strongly agree

**Table 3:** Questions and response options.

Questions	Response Options
Learning outcome	
Q: When you compare the teaching during the COVID-19 lockdown with the teaching before, how do you experience… - Your understanding of what you have been taught? - Your academic development? - Your preparation for exams?	The response options were on a four-point Likert scale: (1) much worse, (2) A little worse, (3) A little better, and (4) much better
Self-regulated learning	
How much do you agree or disagree that during the COVID-19 lockdown you have been able to… - Complete tasks within the specified deadlines? - Plan your time to work on the various tasks? - Know when to have breaks when you lose your concentration? - Avoid procrastination?	The response options were on a four-point Likert scale: (1) strongly disagree, (2) somewhat disagree, (3) somewhat agree, and (4) strongly agree

**Table 4:** Learning gradient and self-regulation. Percent.

	Learning Outcome		Self-Regulation	
	Low Degree of Learning Outcome	High Degree of Learning Outcome	Low Degree of Self-Regulation	High Degree of Self-Regulation
Cluster 1: Prefers 100% online teaching	18.6%	81.4%	27.4%	72.6%
Cluster 2: Mixed (50/50 physical- online)	38.8%	61.2%	54.6%	45.4%
Cluster 3: Prefers physical classroom teaching	67.0%	33.0%	73.1%	26.9%
Significance chi-square	<0.001 Cramer’s V = 0.420 (moderate connection)		<0.001 Cramer’s V = 0.390 (moderate connection)	

Source: Bak and Schulin (2022). From national dataset in 2020 (N = 880).

**Table 5:** Impact of social activities (whether physical or online teaching) on students’ learning outcomes. LEARN 2021 (*n* = 2548). Percent.

Q: Which Form of Teaching Would You Prefer in Your Remaining Study Time?	Impact of Social Activities (Whether Physical or Online Teaching) on Students’ Learning Outcomes.				
	Low Importance	Medium Importance	High Importance	*p*	V
A: I would prefer physical classroom teaching for the rest of my study	4.5%	32.1%	63.4%	<0.001	0.17
A: I would prefer a combination of physical attendance and online teaching for the rest of my study	11.5%	44.2%	44.3%		
A: I would prefer only online teaching for the rest of my study	19.7%	48.4%	32%		

## Data Availability

The collected datasets analyzed during the current study are available from the corresponding author upon reasonable request.
